# Cost effectiveness of preventing falls and improving mobility in people with Parkinson disease: protocol for an economic evaluation alongside a clinical trial

**DOI:** 10.1186/1471-2318-8-23

**Published:** 2008-09-30

**Authors:** Jennifer J Watts, Jennifer L McGinley, Frances Huxham, Hylton B Menz, Robert Iansek, Anna T Murphy, Emma R Waller, Meg E Morris

**Affiliations:** 1 Centre for Health Economics, Monash University, Building 75, 3800 Melbourne, Australia; 2 Murdoch Children's Research Institute, Royal Children's Hospital, 3052 Melbourne, Australia; 3 Clinical Research Centre in Movement Disorders and Gait (National Parkinson Foundation Centre of Excellence), Kingston Centre Southern Health, Melbourne, Australia; 4 Monash Ageing Research Centre, Monash University, 3168 Clayton, Australia; 5 Musculoskeletal Research Centre, Faculty of Health Sciences, La Trobe University, 3086 Melbourne, Australia; 6 Department of Physiotherapy, The University of Melbourne, 3010 Melbourne, Australia

## Abstract

**Background:**

Cost of illness studies show that Parkinson disease (PD) is costly for individuals, the healthcare system and society. The costs of PD include both direct and indirect costs associated with falls and related injuries.

**Methods:**

This protocol describes a prospective economic analysis conducted alongside a randomised controlled trial (RCT). It evaluates whether physical therapy is more cost effective than usual care from the perspective of the health care system. Cost effectiveness will be evaluated using a three-way comparison of the cost per fall averted and the cost per quality adjusted life year saved across two physical therapy interventions and a control group.

**Conclusion:**

This study has the potential to determine whether targetted physical therapy as an adjunct to standard care can be cost effective in reducing falls in people with PD.

**Trial Registration:**

No: ACTRN12606000344594

## Background

Parkinson disease (PD) is a progressive neurodegenerative disorder that mainly affects older individuals. The prevalence rates of Parkinson disease are estimated at 1 per cent in people aged over 60 years and between 0.15 and 0.3 per cent in the general population with a mean age of onset in the mid sixties [1-3]. The burden of disease associated with PD is substantial, impacting on individuals, the healthcare system and society [4].

Hypokinesia, rigidity, rest tremor and postural instability in later stages of the disease are the major motor symptoms of PD [5]. These can progressively restrict mobility and increase the risk of falls [1,5]. Other than motor impairments, PD can affect cognitive function [6] and mood [7]. In some people it can also be associated with dementia [8], sleep alterations, sensory symptoms and autonomic dysfunction [1]. Studies indicate that fatigue, pain and depression are symptoms that have significant impacts on quality of life of people with PD [1,9]. Thus in the latter stages of disease progression PD can be associated with significant disability. Deteriorating functionality and loss of mobility associated with PD typically occur at a time when people are also susceptible to ageing-related changes, compounding these "normal ageing" symptoms [1].

Currently there is no known cure for PD and evidence of effectiveness of neuro-protective agents that slow the progression of the disease is inconclusive [1]. Pharmacotherapy is the most common treatment for motor symptoms although it is reported that complications such as motor fluctuations and dyskinesia are associated with long-term use [1]. A recent systematic review and meta-analysis of the effectiveness of exercise interventions in people with PD found empirical evidence that exercise was beneficial for people with PD with regards to physical functioning, strength and balance and health-related quality of [10].

Annual falls incidence rates in people with PD have been reported to range from 50 – 68 per cent [11-13]. Not only is the risk of falling increased with PD, but the risk of serious falls and falls resulting in injury, particularly hip fracture also increases [14-17]. One study has estimated that 27% of people with PD will sustain a hip fracture in the first 10 years following diagnosis of PD [15]. Predictors of falls include number of falls in the previous year, Hoehn and Yahr stage and 'fear of falling', however the relationship between falls and PD severity combined with level of activity has not been established [17].

A number of costs of illness studies conducted to assess the social and economic burden of PD have shown that PD is costly for individuals, the health-care system and society more broadly [4,18-25]. Drug therapy is a major contributor to direct health care costs [4,21-23], while individuals and their carers also face home care costs and may suffer high productivity losses [4]. Although the costs of falls and fall-related injuries have not been separately analysed in cost of illness studies associated with PD, falls are likely to incur both direct and indirect costs. These include increased costs from health-care service utilization, sustained productivity losses, and impacts on carer quality of life in terms of depression [18], fear for their spouse, and carer injuries sustained while preventing their spouse's falls [19].

A recent New Zealand community-based falls prevention study in an elderly population suggests that falls intervention programs can be cost effective, finding that a home safety program cost $432 per fall prevented [26]. Any intervention that is cost-effective at reducing aspects of the burden of PD, in this case falls, could have important economic benefits for patients and families, and for the health-care system as a whole.

From the perspective of the health system, this prospective economic analysis addresses whether physical therapy is more cost effective than standard care in terms of the number of falls prevented, the number of injurious falls prevented and improvement in health related quality of life (HRQoL). The incremental cost per fall prevented, cost per injurious fall prevented, cost per quality adjusted life year saved are predicted to be less for the two active therapy groups (PRT and MST) than for the control group. No statistically significant difference is predicted between the PRT and MST intervention groups.

## Methods

This study incorporates a prospective economic analysis alongside a randomized controlled trial (RCT). The economic analysis takes the perspective of the health-care system and compares two different physiotherapy programs (movement strategy training (MST) and progressive resistance strength training (PST)) with usual PD care (control group receiving a PD education program without falls education). The three interventions are compared to assess relative cost effectiveness where benefits are measured in terms of two intermediate clinical outcomes, 'falls prevented' and 'injurious falls prevented' and an economic outcome endpoint HRQoL.

### Movement strategy training (MST)

The MST intervention group receives an 8 week outpatient program consisting of a once-weekly physical therapy session (usually provided in groups of 4), which comprises strategies to prevent falls, enhance balance and improve mobility as defined by Morris [27]. Education about risk factors for falls and strategies for reducing falls in the home is included. Participants also complete a home program of exercises once a week. During the 8 weeks each individual also receives one home visit by an occupational therapist (OT), physical therapist or nurse where compliance with the program is monitored.

### Progressive resistance strength training (PRT)

The PRT intervention group receive an 8 week outpatient program consisting of a once weekly 60 minute individualized strengthening program. Resistance is progressively increased and all exercises are functional in design. Participants are also instructed on how to complete the exercises once a week at home. Appropriate equipment including a weighted vest is provided for this purpose. As with the MST intervention, people in the PRT intervention group also receive a home visit.

### Control intervention

The control intervention is an 8 week, once weekly, 60 minute outpatient social activity and PD education program, with an additional activity performed once a week at home. During the program, each person also receives a home visit (as described above).

### Co-interventions

The study does not restrict participants from accessing their usual care or any other activities. It is predicted that a small number of participants might access other physical therapy services, or home modification during the intervention and 12-month follow-up period. The baseline, 3 month and 12 month follow-up questionnaires were designed to capture this information.

#### Study population

Participants are recruited into the RCT from specialist outpatient movement disorder clinics and private neurologists and physical therapists in the Melbourne metropolitan area as well as from advertising in local newspapers. All participants recruited for the RCT are included in the cost effectiveness analysis. To be included, participants are required to have idiopathic PD, be willing and able to provide informed consent, able to walk and safely participate in an exercise program and have the ability to travel to the outpatient clinic for the 8 weeks of therapy and for 4 testing sessions. People are excluded if they scored less than 24 on the Mental State Examination (MMSE) [28], if they are Hoehn and Yahr Stage V [29] (bed or wheelchair-bound), or if they are on major tranquilizers.

#### Sample size

Due to the paucity of trials in people with PD using prospective falls as an outcome measure, the sample size calculation for the study was inherently speculative. Falls rates were initially determined based on data from a previous study of people with PD and older people without PD [30]. The minimum sample size per group required to obtain a statistically significant difference in falls frequency between the control group (with an estimated 60% falls rate) and the PST or MRT groups (with a 40% rate) was calculated as 110 participants (α = 0.05, β = 80%, 10% drop-out rate). However, a recent blinded interim assessment of the falls data indicated a far higher falls rate than initially expected (over 800 falls reported by 93 participants enrolled). An interim analysis by an external data monitoring committee is scheduled in the near future and will consider intervention efficacy, safety and sample size.

#### Blinding

All participants are tested four times by a trained blinded independent assessor: prior to and at the completion of each 8 week intervention, and 3 months and 12 months after completion of the intervention.

#### Ethics Approval

Ethics approvals were obtained from the Southern Health Human Research Ethics Committee (HREC Number 060035) and The University of Melbourne Health Sciences Human Ethics Sub-Committee (Number 0828579) of Australia.

#### Measures of outcomes

An intervention aimed at improving strength or mobility and reducing falls in people with PD is likely to have an immediate effect on both health outcomes and HRQoL. We have therefore incorporated a range of outcome measures aimed at detecting these changes in our core set of fall outcome measures. The primary clinical outcome measure is falls frequency (number of falls per person). A fall has been broadly defined as an unexpected event in which the participant comes to rest on the ground, floor or lower level [31,32]. This definition is consistent with the literature on falls prevention. Changes in HRQoL will be measured using both a PD-specific measure (PDQ-39) [33] and a generic utility instrument (Euroqol-5D) [34] administered at baseline, immediately post-intervention, 3 months, and 12 months.

### Measures of cost

From the health care-system perspective costs incorporate the direct costs of each intervention, including the costs of running the interventions themselves, the costs to the participants of accessing the intervention and any relevant downstream health costs or savings. These costs (and savings) will be quantified, valued, and aggregated to determine total and marginal cost estimates per unit of outcome.

### Direct costs

Direct costs of delivering the interventions will include the resource costs of running the physiotherapy training, the Falls Prevention Education Program, and conducting the home visit. Cost categories will include staff costs (therapists and allied health assistants), equipment (such as weight training equipment used in the PRT intervention), space, capital, overheads, and consumables (education materials). Program development costs will be attributed to each intervention, capturing the costs of training staff to deliver the interventions and developing the intervention protocols.

The costs/savings of health services utilization resulting from events relating to falls and related injuries will be valued to capture the changes in service use associated with reduced falls that result from the interventions. This requires identifying:

• healthcare costs resulting from events relating to falls and related injuries;

• the number and severity of injuries resulting from falls (by injury type);

• the number of hospitalizations and length of stay attributable to falls related injuries; and

• the number of people requiring institutional care following falls injuries.

The direct costs or out-of-pocket payments associated with the interventions that are borne by patients and/or their families will be included. From the health care system perspective the costs of access (transportation) to and from the intervention is likely to be the primary direct personal cost associated with the intervention.

### Indirect costs

Indirect costs associated with the interventions will be included in the analysis. These are the opportunity cost for participants and their carers of the intervention (patient time), and productivity gains/losses in work and leisure that result from the intervention.

### Exclusion of trial costs

All setup costs and other costs related to the clinical trial will be excluded. These include costs associated with trial administration, data collection, and actual measurement of clinical outcomes as per the trial protocol.

## Results

### Outcome data

**Figure 1:**
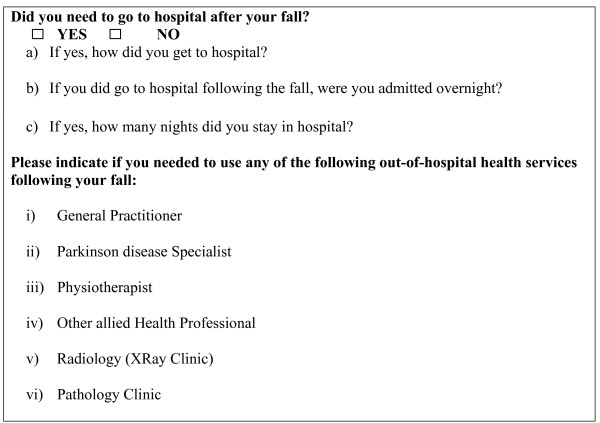
Economic data/questions included on the Falls Questionnaire.

A falls incidence reporting system has been designed to collect comprehensive falls data, comprising a falls calendar, a falls telephone hotline, and regular 'falls-specific' calls to frequent 'fallers' for clarity and completeness [32]. The questions relating to economic data associated with each fall are shown in Figure 1. Following a fall, participants record the event on an especially designed calendar and telephone the 'Falls Hotline'. A standardized questionnaire is administered by a trained assistant when a participant reports a fall through the hotline or in a follow-up phone call for falls reported through the falls calendar. This questionnaire collects important sub-classification data on the type and cause of the fall and includes a comprehensive set of questions on any health service use arising as a result of the fall.

Falls are classified as injurious and non-injurious falls. Detailed falls classification data are also collected through the falls follow-up standardized questionnaire on injuries sustained. Hospitalization information including length of stay, transport mode to hospital, and details of the hospital, together with other out-patient health care service utilization (general practitioner, Parkinson's specialist, physiotherapist, other allied health professional, radiology clinic and pathology clinic) are collected. This latter information will also be used to determine the extent of injury associated with falls, to define falls as 'injurious' or 'non-injurious' and the associated costs of falls.

Making this distinction between injurious and non-injurious falls and the data collection strategy developed will reduce misclassification and reporting errors [31]. Further, from a health-care system perspective this distinction is beneficial in providing more specific and deeper analysis of both the effectiveness and health-care costs/savings associated with the interventions.

Quality of life data is obtained from the two Quality of Life surveys (the PDQ-39 questionnaire that collects disease-specific quality of life data and the Euro-QOL which generates a generic health quality of life utility measure), administered to each participant at the baseline, post-intervention, 3 months, and 12 month interview.

Changes in disability attributable to the intervention are measured using the Unified Parkinson's Disease Rating Scale (UPDRS) [35]. This will enable us to stratify falls by the level of disability.

### Cost data

A questionnaire administered to facility managers will quantify the resource requirements, including staff costs, rent for space and large capital items, overheads and consumables. Market prices and relevant depreciation rates will be used to determine other capital and equipment costs. Sensitivity analysis will be used to vary labour costs using the award wage rate plus on-costs for superannuation and leave loading, and market fee-for-service charges for the relevant health professional category.

A questionnaire, to be administered at each therapy session, has been designed to capture information on any injuries or health issues and associated healthcare utilisation arising as a direct result of the therapy sessions. Personal costs borne by the individual in accessing the intervention including time, distance and mode of transport are also included. Costs of each mode of transport will be estimated using standard taxi charges, the price of public transport, and a cost per kilometre for a private car.

Data collected through the falls calendar and falls hotline will be used to quantify fall-related health care service utilization. Valuing or costing health service utilization will be done using a number of methods. Where patients attend a hospital in our Southern Health region (which includes hospital facilities participating in the study), we will collect patient-level costing and utilization data for inpatient services from the hospital's computerized costing and admission discharge systems. This data can then be used to model costs for patients where patient-level data is not available, such as if they attend a hospital outside the Southern Health region. In this case length of stay will be used as the main parameter. Costs will be attributed to these services based on published costing data from the National Hospital Cost Data Collection, Commonwealth Medical Benefits Schedule (CMBS), and the Pharmaceutical Benefits Scheme (PBS). This approach can be reconciled with a Diagnosis Related Group (DRG) costing approach. CMBS rates will be used to cost medical and diagnostic imaging services, PBS for pharmaceuticals and award wages for other health professionals.

#### Cost effectiveness analysis

The benefit of economic evaluation is that it captures both benefits and costs of an intervention in one comparable unit – a cost-effectiveness ratio. In this evaluation economic analysis will be three-way, first comparing each intervention with the control group, and then comparing the interventions with each other. The three-way analysis of the cost effectiveness ratios is shown in Table 1.

**Table 1:** Three-way analysis of cost-effectiveness ratios

**MST vs Control**		
Cost per fall averted	Cost per injurious fall averted	Cost per QALY saved

**PRT vs Control**		

Cost per fall averted	Cost per injurious fall averted	Cost per QALY saved

**MST vs PRT**		

Cost per fall averted	Cost per injurious fall averted	Cost per QALY saved

### Cost per fall averted

Increased risk of falling is a significant adverse consequence of PD. As such, reduced falls is the primary clinical outcome of the RCT. The first step of the economic evaluation will be to analyse this intermediate outcome, and do a three way comparison of the cost per fall averted ratios of the interventions and the control. This ratio provides a crude measure of cost effectiveness in that the outcome measure (fall averted) does not distinguish between different levels of severity of falls. However as a standardised definition it enables greater synthesis with the body of research in the field and allows comparison with other falls prevention studies [31].

### Cost per injurious fall averted

Falls are broadly defined in this trial, consistent with best practice in the field [31,32]. However there is significant variation in the severity of falls and the associated direct and indirect costs of falls (injury, quality of life consequences, hospital and other medical costs of treatment, and productivity losses). One intervention may be more effective at reducing serious falls (resulting in injury) while another may be more effective at reducing minor falls. An intervention may also improve the ability of a person with Parkinson's disease to react, hence minimising the severity of a fall and changing the type of fall endured. While cost of hospital use associated with falls is captured in the first ratio (primary outcome measure), from a health services perspective it is also meaningful to differentiate between falls and analyse more specifically falls associated with injury and subsequent health care use. We will therefore define an 'injurious fall' as a fall resulting in an injury requiring a health service contact.

### Cost per quality adjusted life year saved

Falling and fear of falling can reduce the quality of life of individuals with PD in a number of ways. Further it is expected that physiotherapy treatments may have other positive impacts on individuals with PD. Comparing interventions based only on their ability to prevent falls will not capture the broader impacts and quality of life changes that may be attributable to the interventions. There is also limited external comparability of the cost effectiveness of these interventions with other interventions for people with PD, let alone other diseases. Thus cost utility analysis will be undertaken comparing the cost per QALY gained across the two interventions and the control group.

#### Discounting and sensitivity analysis

We will define our base year as 2008 so costs in earlier years will be inflated using the published Australian consumer price index (CPI). One way sensitivity analysis will be undertaken to investigate the robustness of the CE ratios to a range of cost and effect estimates, including staff and program costs and discount rate on the cost side, and number of falls, number of injurious falls, HRQoL and PDQ-39 on the effect side.

## Discussion

There are a number of important advantages of conducting an economic evaluation alongside an RCT, especially where the trial compares complex interventions for a disease such as PD. Economic input throughout the planning, design, data collection, implementation and evaluation and analysis phase of the RCT means that data can be collected alongside the clinical data in a way that meets the specific needs of the economic analysis. A central database system can be used to store both clinical and economic data concurrently. Timely data collection means that recall bias is reduced.

Inclusion of an economist in the design and running of the RCT results in expert advice regarding the selection of appropriate outcome measures. In this evaluation, there is opportunity to access good quality effectiveness data through careful monitoring of falls outcomes and timely reporting on quality of life indicators in the same sample population. Consistency in applying the outcome measurement instruments to the same sample population may not be available in evaluations using modelling and published findings.

The clinical trial setting provides more specific, and sometimes patient-level, resource use and costing data involved in running the interventions, where estimates can be modelled from the costs incurred during the trial itself. Further, more systematic and timely consideration of costs can take place in this planned environment.

There are potentially some limitations to this methodology. Clinical trials are conducted in natural settings, so treatment compliance, resource utilization and other outcomes observed in a trial may not reflect what actually occurs in the real world. Using more real-life setting observational data or more pragmatic clinical trials could improve the external validity of an economic evaluation attached to a clinical trial. In this trial, the intervention protocols were designed to proximate real-life environments by running the interventions through out-patient facilities.

The reporting system for falls established for this RCT and evaluation is made up of two data capture mechanisms, the calendar and the falls hotline, which allows for cross-checking of falls reports. This appears to improve reporting rates of fall events and track compliance of the reporting mechanism. The standardized questionnaire, administered when contact is made through the falls hotline and/or during a follow-up phone call for calendar reported falls, is designed to reduce recall bias by reducing the recall period (maximum of 1 month for falls reported on the calendar that were not reported through the falls hotline) and by systematically documenting fall events and their implications, particularly with respect to health service use.

Two measures of quality of life have been chosen for this study, the EuroQoL EQ-5D and the PD-specific PDQ-39. These tools were chosen due to their simplicity for respondents, and the documented validity, reliability and sensitivity to change in the quality of life literature [32,34,36]. The PDQ-39 captures the specific impacts on quality of life that relate specifically to people with PD. The single unitary health utility index derived from the EQ-5D will enable comparison across a range of interventions, a broad scope of health issues and diseases, and may also capture "unanticipated effects" not built into the PDQ-39 [36,37].

## Conclusion

This project has the potential to determine whether physical therapy as an adjunct to the routine medical management of PD can be cost effective in reducing falls and improving quality of life. Evidence of cost effectiveness can be used to support a case to healthcare funders, including private insurers for the funding of a complex physical therapy intervention for people with this disabling neurological condition.

## Competing interests

The authors declare that they have no competing interests.

## Authors' contributions

MM, FH and JM conceived the idea for the study and participated in the design of the study as well as project management, data analysis and data interpretation. JW conceived the idea for the economic evaluation component of the study, participated in the design of the study, and drafted the manuscript for submission to *BMC Geriatrics*. AM and RI contributed to the design and assisted with subject recruitment and safety monitoring. HBM contributed to the design of the study, especially the falls data collection and falls analysis. EW assisted in drafting the manuscript for submission to *BMC Geriatrics *and is involved in compiling patient information. All the authors have read and approved the manuscript.

## Pre-publication history

The pre-publication history for this paper can be accessed here:


